# RETRACTION: Evaluating a Technologically Enhanced Rehabilitation Programme for Wound Gealing in Patients with Coronary Heart Disease

**DOI:** 10.1111/iwj.70479

**Published:** 2025-04-02

## Abstract

**RETRACTION:** HongF. , LiuF. , LiY. , and LiuP. , “Evaluating a Technologically Enhanced Rehabilitation Programme for Wound Gealing in Patients with Coronary Heart Disease,” International Wound Journal 21, no. 4 (2024): e14568, 10.1111/iwj.14568.38124400 PMC10961874

The above article, published online on 20 December 2023, in Wiley Online Library (http://onlinelibrary.wiley.com/), has been retracted by agreement between the journal Editor in Chief, Professor Keith Harding; and John Wiley & Sons Ltd. Following an investigation by the publisher, all parties have concluded that this article was accepted solely on the basis of a compromised peer review process. The editors have therefore decided to retract the article. The authors did not respond to our notice regarding the retraction.

**RETRACTION:** F. Hong , F. Liu , Y. Li , and P. Liu , “Evaluating a Technologically Enhanced Rehabilitation Programme for Wound Gealing in Patients with Coronary Heart Disease,” International Wound Journal 21, no. 4 (2024): e14568, 10.1111/iwj.14568.38124400 PMC10961874

The above article, published online on 20 December 2023, in Wiley Online Library (http://onlinelibrary.wiley.com/), has been retracted by agreement between the journal Editor in Chief, Professor Keith Harding; and John Wiley & Sons Ltd. Following an investigation by the publisher, all parties have concluded that this article was accepted solely on the basis of a compromised peer review process. The editors have therefore decided to retract the article. The authors did not respond to our notice regarding the retraction.

